# Intra-Arterial Chemotherapy as Primary Therapy for Retinoblastoma in Infants Less than 3 Months of Age: A Series of 10 Case-Studies

**DOI:** 10.1371/journal.pone.0160873

**Published:** 2016-08-09

**Authors:** Miaojuan Chen, Junyang Zhao, Jiejun Xia, Zhenyin Liu, Hua Jiang, Gang Shen, Haibo Li, Yizhou Jiang, Jing Zhang

**Affiliations:** 1 Department of Interventional Radiology and Vascular Anomalies, Guangzhou Women and Children’s Medical Center, Guangzhou 510623, China; 2 Department of Ophthalmology, Beijing Children’s Hospital, Capital Medical University, Beijing, China; Massachusetts Eye & Ear Infirmary, Harvard Medical School, UNITED STATES

## Abstract

**Purpose:**

Retinoblastoma is the most common primary malignant intra-ocular tumor in children. Although intra-arterial chemotherapy (IAC) by selectively infusing chemotherapy through the ophthalmic artery has become an essential technique in the treatment of advanced intra-ocular retinoblastoma in children, the outcome of IAC as primary therapy for infants less than 3 months of age remains unknown. In this retrospective study, we reviewed the outcome of IAC as primary therapy for retinoblastoma in infants less than 3 months of age.

**Methods:**

We retrospectively reviewed ten retinoblastoma patients attending our center from January 2009 to September 2015 and beginning primary IAC before the age of 3 months. The patient characteristics, overall outcomes and therapy-related complications were assessed.

**Results:**

The mean patient age at the first IAC treatment was 10.4 weeks (range 4.9–12.9 weeks). These eyes were classified according to the International Classification of Retinoblastoma (ICRB) as group A (n = 0), B (n = 2), C (n = 0), D (n = 9), or E (n = 2). A total of 28 catheterizations were performed, and the procedure was stopped in one patient because of internal carotid artery spasm. Each eye received a mean of 2.6 cycles of IAC (range 2–4 cycles). After IAC with a mean follow-up of 28.3 months (range 9–65 months), tumor regression was observed in 12 of 13 eyes. One eye was enucleated due to tumor progression. All patients are alive and no patient has developed metastatic disease or other malignancies.

**Conclusions:**

Our experience suggests IAC as primary therapy is a feasible and promising treatment for retinoblastoma in infants less than 3 months of age.

## Introduction

Retinoblastoma (RB) is the most common intra-ocular malignant tumor in infants and young children, affecting approximately 1 in 16000–18000 live births worldwide each year [1, 2]. In the past, external beam radiotherapy and enucleation were considered as the best options for advanced retinoblastoma [3, 4]. However, the management of retinoblastoma has evolved significantly over recent years. The current treatment strategies not only save the patient’s life, but also preserve globe and vision, even minimize complications or side effects of treatment. Therefore, the treatment of retinoblastoma has shifted away from eye enucleation and external radiation towards chemotherapy plus local treatment [5, 6]. The application of systemic chemotherapy and local treatment greatly increases survival rate and reduces complications, but insufficient efficacy to refractory and advanced retinoblastoma tumors and concerns about short and long-term toxicity of intravenous chemotherapy (IVC) led clinicians to intra-arterial chemotherapy (IAC) by selectively infusing chemotherapy through the ophthalmic artery using modern microcatheters [7–10]. Because IAC could significantly reduce the enucleation and minimize the toxicity of systemic chemotherapy, it has become the first-line treatment for advanced intra-ocular retinoblastoma.

Approximately 1100 to 1500 new cases of retinoblastoma are diagnosed every year in China. Most patients with retinoblastoma present themselves at an advanced stage without timely diagnosis and appropriate treatment, as Huang reported the average age at first diagnosis was 2.2 ± 1.7 years and 81.47% were D-E stages of retinoblastoma [11–14]. Since we realized that IAC is effective, has acceptable ocular complications and minimal systemic toxicity, we mainly use IAC as primary therapy for advanced intra-ocular retinoblastoma at our center. Although many studies have reported that IAC is a safe and effective treatment option for patients with advanced retinoblastoma, there is limited information regarding the outcomes of IAC as primary therapy for retinoblastoma in infants less than 3 months of age, which is why we think it is meaningful to report our experience. In this study, we retrospectively reviewed the outcome of IAC as primary therapy for retinoblastoma in infants less than 3 months of age.

## Methods

We obtained approval for this retrospective study from the ethics committee of Guangzhou Women and Children’s Medical Center. Written informed consent was obtained from patients, carers or guardians on behalf of all children enrolled in this study, and the associated risks were explained in detail. Based on the review of medical records, we retrospectively analyzed patient records from January 2009 to September 2015 from our center to select patients fulfilling the following eligibility criteria: patients with retinoblastoma received primary IAC for the management of retinoblastoma eye and were less than 3 months of age at first IAC treatment.

Eyes were examined under anesthesia by the ocular oncologist. Fundus examination was performed with Retcam fundus camera. After fundus examination, the decision to treat with IAC was undertaken in consultation with the retinoblastoma treatment team. Inclusion criteria for the use of IAC were patients with advanced intra-ocular retinoblastoma in at least one eye (Group D or E). Patients were excluded if the retinoblasoma could be controlled with focal treatments (laser, cryotherapy, or brachytherapy), was at a vary advanced stage (neovasular glaucoma or suspected invasion of postlaminar optic nerve, sclera, or anterior chamber) or if they had extraocular invasion, documented metastatic disease or structural brain abnormalities. With patients under general anesthesia, one femoral artery (usually right) was punctured and a 3F or 4F arterial sheath was placed into the femoral artery. 75 IU/kg of heparin was administered intravenously to achieve systemic anticoagulation. The arterial anatomy was visualized with serial angiography runs and the ostium of the ophthalmic artery was superselectively catheterized with a microcatheter. The chemotherapeutic agents used in the protocol included melphalan, topotecan, or carboplatin. The dose of melphalan was not more than 0.5 mg/kg. Topotecan dose was 0.5–1 mg and carboplatin dose was 20–30 mg. The dose of chemotherapeutic agent was adapted according to the response from the previous procedure of IAC. Melphalan or topotecan was diluted with 0.9% saline and carboplatin was diluted with 5% glucose injection, which were administered in a pulsatile fashion over 30 minutes. After the procedure, the catheter and sheath were removed and hemostasis of the femoral artery was obtained with manual compression. The patient was monitored overnight before discharge. A complete blood count was performed every three days for two weeks after the procedure. The middle meningeal artery technique was used as an alternative for eyes that failed ophthalmic artery catheterization. Each cycle of IAC was performed at a 4-week interval, and the necessity for further sessions was decided based on tumor response. Detailed ophthalmic examination was performed at each 4-week follow up by the ocular oncologist, who was blinded to the aim of this study. During follow-up, each eye was assessed for regression of solid tumor, subretinal seeds, vitreous seeds, and subretinal fluid. Tumor recurrence was documented. Other subsequent ocular treatment was recorded.

The patient records were retrospectively reviewed for demographic data [age (months), sex (male, female), family history, and laterality (unilateral, bilateral), weight and age at first IAC, other treatment (laser, enucleation, IVC), and follow-up duration]. Tumor data included International Classification and response to treatment. The eyes were classified according to the University of California Los Angeles version of the International Classification of Retinoblastoma (ICRB) as group A, B, C, D, or E [15].

## Results

### Patient and eye characteristics

**Fig 1:**
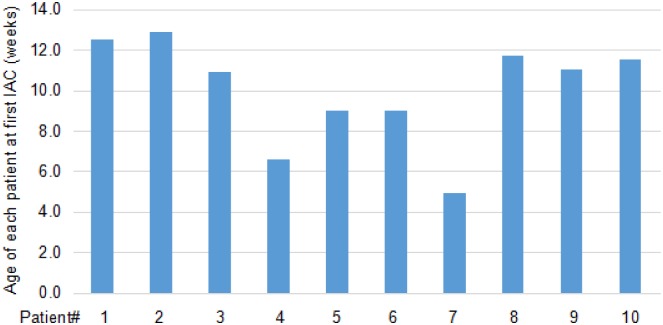
The age of each patient at first IAC treatment.

A total of 13 eyes of 10 patients fulfilled the inclusion criteria from January 2009 till September 2015. All of them did not receive prior treatment and were treated with IAC as primary therapy. Table 1 summarizes patient, eye, treatment, and outcome characteristics. There were 6 females and 4 males. The mean patient age at diagnosis was 7.9 weeks (range 4.6–10.9 weeks) and at first IAC treatement it was 10.4 weeks (range 4.9–12.9 weeks) (Fig 1). Nine patients presented with leukokoria at the time of diagnosis, and one patient was found in cataract screening. The fundus examination revealed that seven patients displayed unilateral and three displayed bilateral disease. The 13 eyes were classified as ICRB group B (n = 2), group D (n = 9), and group E (n = 2) at the first visit. Each patient had at least one Group D or worse eye, necessitating IAC. None of the patients had family history.

**Table 1:** Results of IAC for retinoblastoma in infant less than 3 months of age.

N	Sex	Uni/Bi	Family history	Signs	Age & weight at First IAC	Eyes undergoing treatment	Classification: ICRB	No. IAC cycles	Other treatment	Response: Tumor regression	Follow-up (months)	Complication
1	F	U	no	leukocoria	12.5 weeks & 7 Kg	OS	E	3	none	Type 1	9	no
2	F	B	no	leukocoria	12.9 weeks & 6.2 Kg	OS	D	4	none	Type 1	18	vitreous hemorrhage
						OD	B	4	laser	Type 4	18	no
3	F	U	no	leukocoria	10.9 weeks & 7 Kg	OS	D	3	none	Type 1	10	vitreous hemorrhage
4	M	U	no	leukocoria	6.6 weeks & 7.5 Kg	OS	D	3	none	Type 1	14	no
^1^5	M	U	no	leukocoria	9.0 weeks & 6.0 Kg	OS	E	2	IVC	Type 1	9	phthisis, ophthalmic artery spasm, neutropenia
6	M	U	no	none	9.0 weeks & 7 Kg	OD	D	2	laser	Type 1	47	choroidal infarction
7	F	U	no	leukocoria	4.9 weeks & 6.8 Kg	OS	D	3	none	Type 1	26	no
8	M	B	no	leukocoria	11.7 weeks & 9.8 Kg	OS	D	2	none	Type 1	19	ptosis, phthisis
						OD	B	2	laser	Type 4	19	no
9	F	B	no	leukocoria	11.0 weeks & 6 Kg	OS	D	2	enucleation+ IVC	n/a	65	eyelid edema
						OD	D	2	laser	Type 4	65	no
10	F	U	no	leukocoria	11.5 weeks & 7 Kg	OS	D	2	none	Type 1	49	phthisis

IVC: Intravenous chemotherapy; IAC: Intra-arterial chemotherapy; F: female; M: male; Uni: unilaterality; Bi: bilaterality; OD: Oculus Dexter; OS: Oculus Sinister.

Tumor regression pattern: Type 0: disappearance; Type 1: highly calcified; Type 2: fish flesh (no vascularity and no growth); Type 3: mixed types 1 and 2; Type 4: white scar.

^1^The only catheterization failure was the second cycle of IAC in Patient #5

### Treatment

Treatment features are shown in Table 1. IAC was performed in 13 eyes of 10 patients (bilateral in 3 cases), with a median of 2.6 cycles per eye (range 2–4), and 3.4 cycles per patient (range 2–4). A total of 28 catheterizations were performed, and the procedure was stopped in one patient because of internal carotid artery spasm. Of 27 successful catheterizations (96%), the chemotherapeutic agent was infused directly into the ophthalmic artery, except for once, in which the case the agent was infused into the ophthalmic artery using the middle meningeal artery. The only catheterization failure occurred in the second cycle of IAC in Patient #5. The chemotherapeutic agents used were melphalan (n = 27) plus either topotecan (n = 16) or carboplatin (n = 11).

Two patients (Patient #5 and #9) received IVC with carboplatin, vincristine and etoposide. Patient #5 was successfully treated with the first cycle of IAC as primary therapy, and the fundus examination showed that tumor regression happened after the first cycle of IAC (data not shown). Patient #5 was treated with the second cycle of IAC, but the procedure was stopped because of internal carotid artery spasm. Then Patient #5 had to receive IVC for the treatment of retinoblastoma. Patient #9 was treated with IAC as primary therapy after diagnosis. After two cycles of IAC, fundus examination showed tumor regression observed in the right eye of Patient #9 was observed, even though adjuvant laser consolidation was then required for the small tumor. However, the left eye of Patient #9 was enucleated for progression of disease after two cycles of IAC, and the patient was treated with IVC as adjuvant for the enucleated left eye. Four patients (Patient #2, 6, 8 and 9) required laser therapy for the treatment of small tumor after IAC. In the follow-up period, if recurrence of intraretinal tumor was small (less than 3 mm in any diameter and no vitreous seeding), patients would be treated with laser.

### Complications

The treatment complications are listed in Table 1. A total of 7 patients had complications. One patient (Patient #5) had catheter-induced ophthalmic artery spasm during IAC. One patient (Patient #5) had Grade 3–4 neutropenia and then received a red blood cell transfusion. Ocular complications included vitreous hemorrhage in two eyes, phthisis in three eyes, ptosis in one eye, eyelid edema in one eye, and choroidal infarction in one eye. In four patients (Patient #1, 4 and 7), no complication was reported. There were no patients with fever, sepsis and other systemic toxic effects.

### Clinical results

**Fig 2:**
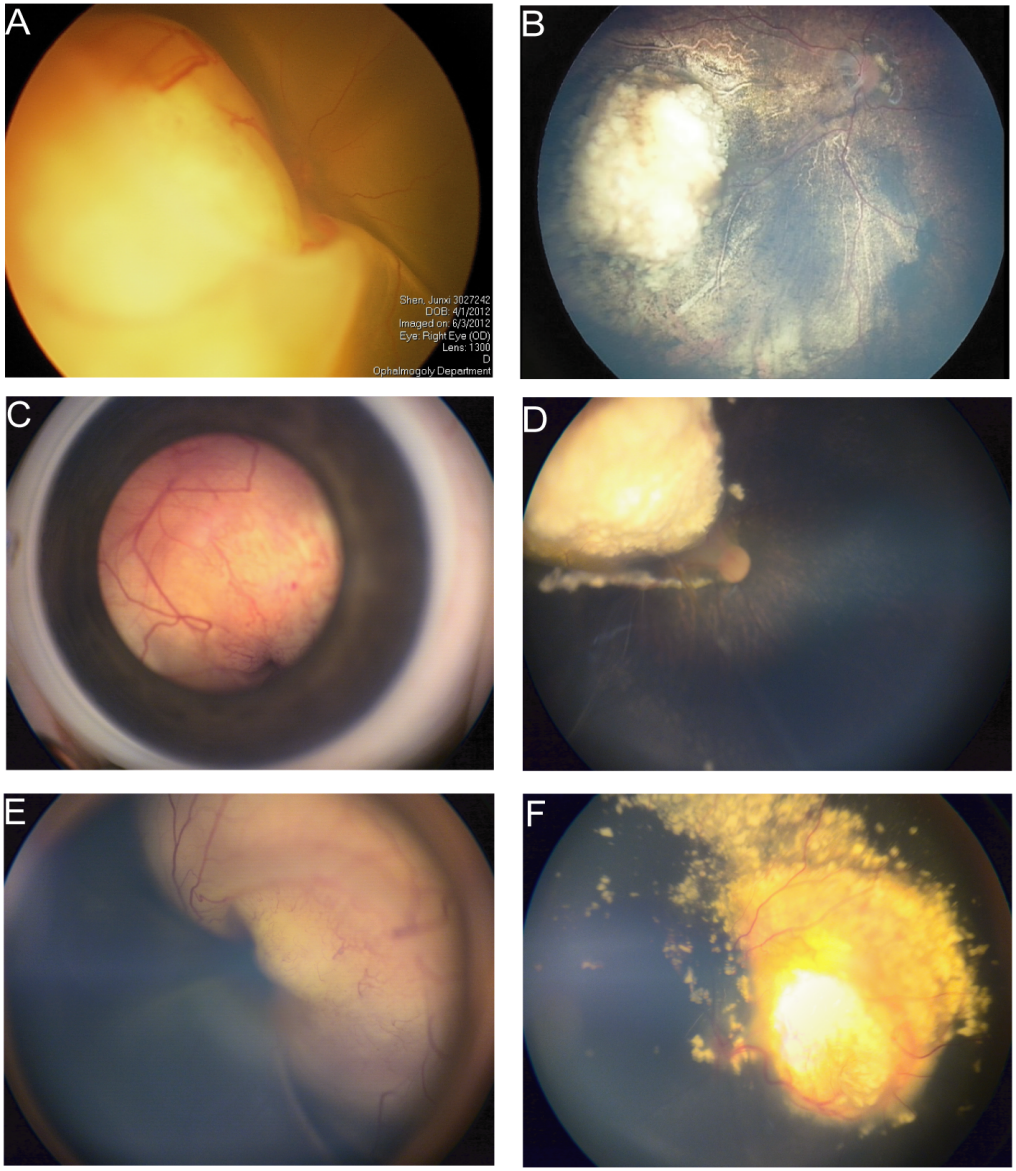
Intra-arterial chemotherapy (IAC) as primary therapy for infants less than 3 months of age. A&B: 6.9-week-old boy with unilateral sporadic retinoblastoma in right eye (Group D). A, Fundus photographs at initial examination (6.9-week-old). Right eye: large tumor with retinal detachment. B, Fundus photographs after 2 cycles of intra-arterial chemotherapy. After 2 cycles of IAC, complete tumor regression was achieved. C&D: 6.6-week-old boy with unilateral sporadic retinoblastoma in left eye (Group D). C, Fundus photographs at initial examination (6.6-week-old). Left eye: large tumor with retinal detachment. D, Fundus photographs after 3 cycles of intra-arterial chemotherapy. The tumor has shrunk and highly calcified. E&F: 11.3-week-old girl with unilateral sporadic retinoblastoma in right eye (Group D). E, Fundus photographs at initial examination (11.3-week-old). Right eye: large tumor with retinal detachment. F, Fundus photographs after 3 cycles of intra-arterial chemotherapy. The tumor has shrunk and highly calcified.

All patients are alive and none have developed extra-ocular disease or a second cancer at a mean follow-up of 28.3 months (range 9–65 months). The clinical results are detailed in Table 1. The fundus examination showed that tumor regression was observed in 12 of 13 eyes. One eye was enucleated (patient #9) due to progression of disease. Tumors in 5 eyes of 5 different patients completely regressed after two or three cycles of IAC, and no additional therapy was required. Fig 2 is the examples of treatment.

## Discussion

In recent years, many specialized centers across the world have used IAC, and this is now widely considered a safe and effective method of management of advanced intra-ocular retinoblastoma [16–19]. However, there are few reports regarding the outcome of IAC as primary therapy for retinoblastoma in infants of less than 3 months old [20]. In this study, we retrospectively analyzed 10 retinoblastoma patients who began IAC within their first 3 months of life (range 4.9–12.9 weeks). The clinical results showed that primary IAC is feasible for the treatment of retinoblastoma in that age group. Although most (12/13) eyes were salvaged, the follow-up period was short (three patients with less than one year follow-up). It is difficult to evaluate the long-term efficacy and safety of IAC. Therefore, longer follow-up is necessary and continued follow-up is ongoing.

In China, most eyes with retinoblastoma are always at a relatively advanced clinical stage of disease (Group D or E) when the patients present to hospital. Among these patients, some were less than 3 months of age. Treatment of infants less than 3 months of age with retinoblastoma is especially challenging [21, 22]. In our center, at first, the patients less than 3 months of age with advanced intra-ocular retinoblastoma were treated with primary systemic chemotherapy. Sometimes, enucleation is required due to the progression of disease. However, Although IVC is effective in shrinking the tumors when performed in combination with local treatment, it is less effective in tumors diagnosed at less than 3 months of age, which agreed with the result from Gombos study [23]. Poor response to chemotherapy, higher frequency of systemic side effects with IVC and its potential for future hospitalization have led our group to an alternative therapy as primary therapy for patients less than 3 months of age [24–26]. With the limited systemic side effects profile and favorable outcomes, IAC is used as primary therapy for infants less than 3 months of age instead of systemic chemotherapy in our center. Although Yamane reported a case of IAC performed in a patient as young as one month and 5 days in his series of 187 patients and Magan reported a successful case of IAC performed in a 2-month-old infant [27, 28], no other papers report the outcome of IAC as primary therapy for retinoblastoma in infants less than 3 months of age, which is why we think it very important to share our experience.

The current drug of choice for IAC (melphalan) may induce significant neutropenia when given at a dose higher than 0.5 mg/kg [29]. Therefore, firstly, drug dosage was calculated on the basis of a patient’s age, which provides an estimate of the eye size and angioanatomy, rather than his/her body weight [30]. Body weight is only used for limiting the total systemic dose in the youngest children with bilateral treatment, especially with melphalan [10]. Coincidentally, all patients in this cohort had reached 6 kg when they received the first cycle of IAC. So the drug dose was adjusted from the standard dose as previously described according to clinical and anatomical factors and ranged from 2.5–3 mg for melphalan. Secondly, we used double drug IAC for using smaller doses of melphalan by combining with carboplatin or topotecan to avoid the system toxicity of single drug chemotherapy. Melphalan 2.5–3 mg is the first choice and is supplemented with topotecan 0.5–1 mg or carboplatin 20–30 mg. Lastly, to achieve the best effect without serious complications, the dosage of chemotherapeutic agent was adapted according to the response to the previous IAC cycle. In this study, after treatment with IAC, 10% of patients had Grade 3–4 neutropenia, which was similar to the results from the older patients [28]. Abramson and associates reported that bridge IV-IA chemotherapy was feasible and safe, and is a promising strategy to treat retinoblastoma in neonates and young infants [20]. They used IVC to postpone IAC until children reach the age of 3 months and a weight of 6 Kg. After retrospectively analysis, we found that the recovery and the complication of IAC as primary therapy for retinoblastoma in in infants less than 3 months of age were similar to those of IAC during bridge IV-IA chemotherapy. As for the eyes that developed the pthisis (Patient #5 OS, Patient #8 OS, Patient #10 OS), this could be secondary to other pathological changes (e.g. glaucoma) that caused optic nerve atrophy and then pthisis bulbi before IAC, which needs to be further confirmed.

IAC requires repeated placements of an arterial sheath in the femoral artery and catheterizations of the ophthalmic artery. The femoral artery is the most preferred access site for therapeutic catheterization procedure [31, 32]. However, in infants less than 3 months of age, the femoral artery is just slightly larger than the catheters. Catheterization might not be successful or may result in complications. Therefore, whether the femoral artery of infants less than 3 months of age is an issue during IAC remains uncertain, even though Yamane and Magtan had reported that IAC was successfully performed for the treatment of retinoblastoma in young infants [27, 28]. In this study, no complications happened in the patients, such as arterial thrombosis or dissection. After IAC and at review every three months, we found that there were no differences in the temperature and the femoral pulse between the lower limbs and no abnormalities in lower limb function has been noted. These observations further strengthen the evidence that IAC is feasible for infants less than 3 months of age. In addition, young children inevitably receive repeat general anaesthesia during IAC. Most parents’ concerns are whether general anaesthesia in infancy affects brain development. A recent report showed that sevoflurane general anaesthesia in infancy does not result in substantial neurotoxicity, which should ease the public concerns over general anaesthesia [33]. Therefore, IAC seems to be safe for infants less than 3 months of age.

In conclusion, although these results are preliminary and longer follow-up is necessary, our results confirm that primary IAC is feasible in infants less than 3 months of age and justify further investigation of its use as primary therapy for retinoblastoma in this context.
